# Epithelioid Angiomyolipoma of Liver with an Inflammatory Component: A Case Report

**DOI:** 10.1155/2013/738708

**Published:** 2013-03-10

**Authors:** Jyothi C. R., Dhanya P. Menon, Joy Augustine, A. K. Abdul Siyad

**Affiliations:** ^1^Department of Pathology, Government Medical College, Thrissur 680596, India; ^2^Department of General Surgery, Government Medical College, Thrissur 680596, India

## Abstract

Angiomyolipomas (AMLs) are benign mesenchymal tumors seen in kidneys in association with tuberous sclerosis. They are uncommon in liver. Angiomyolipomas of liver show great histological diversity and various types and patterns are described. Among them, epithelioid and inflammatory angiomyolipomas are rare. We report a case of epithelioid angiomyolipoma of Liver with an inflammatory component.

## 1. Introduction

 Angiomyolipomas are benign mesenchymal tumors seen in the kidney either sporadically or in association with tuberous sclerosis [1]. They belong to the perivascular epithelioid cell tumor family and coexpress melanocytic and smooth muscle cell markers [2]. Hepatic angiomyolipomas were first described by Ishak in 1976 [3]. Only 200 cases have been reported in the English literature so far [4]. They are seen most commonly in adult females [5] and are located in the right lobe of liver [6]. These lesions are difficult to diagnose by imaging studies, especially when the fat component is scant or absent [7]. Because of their epithelioid morphology, they may be mistaken for hepatocellular carcinoma [8]. Presence of inflammatory cells in epithelioid angiomyolipoma is rare, and it may resemble an inflammatory myofibroblastic tumor [9]. Immunohistochemistry is useful for diagnosis as the smooth muscle cells are positive for HMB45 and SMA [10].

## 2. Case Report

**Figure 1:**
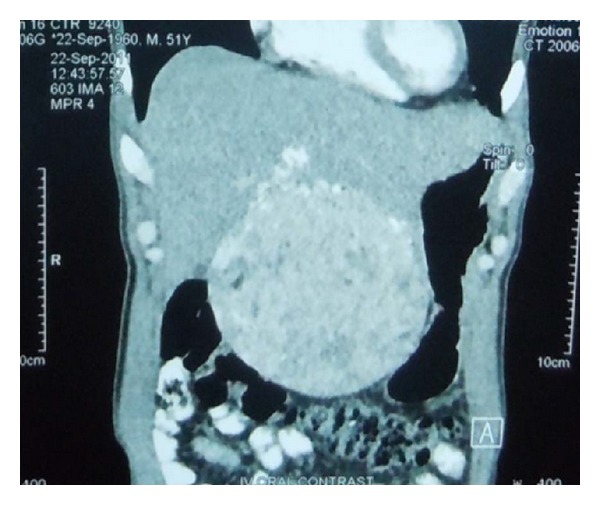
CT liver showing a mass lesion.

A 50-year-old gentleman came to the casualty with a history of fever, vomiting, and abdominal pain. He was a nonsmoker and nonalcoholic with no history of diabetes mellitus or hypertension. The patient did not have any features of tuberous sclerosis complex. An ultrasonogram showed a mass in the liver. CT showed a large mass in the left lobe of liver suggestive of hepatocellular carcinoma or giant angioma (Figure 1). The patient underwent lateral segmentectomy of liver.

**Figure 2:**
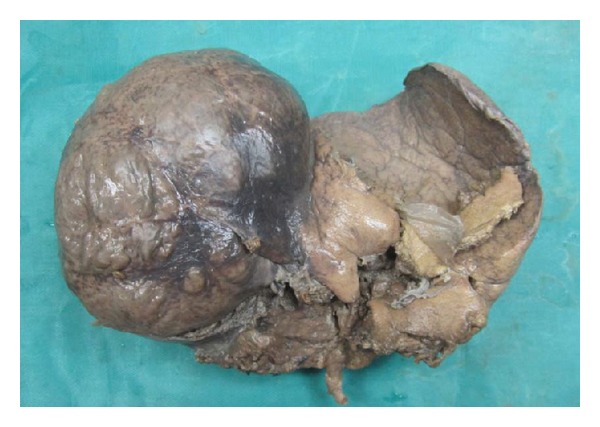
liver with a cystic lesion.

**Figure 3:**
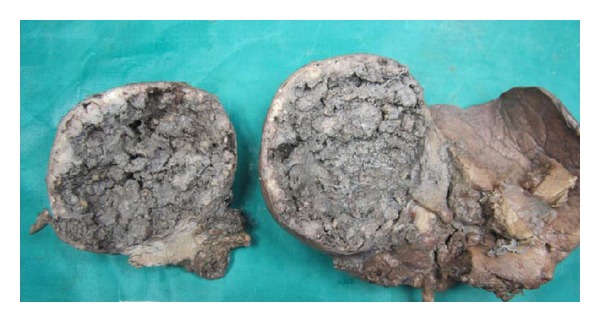
c/s of lesion—brownish, haemorrhagic, and necrotic.

We received segmentectomy specimen of liver which weighed 650 grams and measured 15 × 9 × 8 cms. Surface was bosselated (Figure 2). Cut section showed a well-encapsulated cystic mass measuring 8 cms in diameter. Cut surface showed necrotic and hemorrhagic appearance with grey white firm areas in the periphery (Figure 3). 

**Figure 4:**
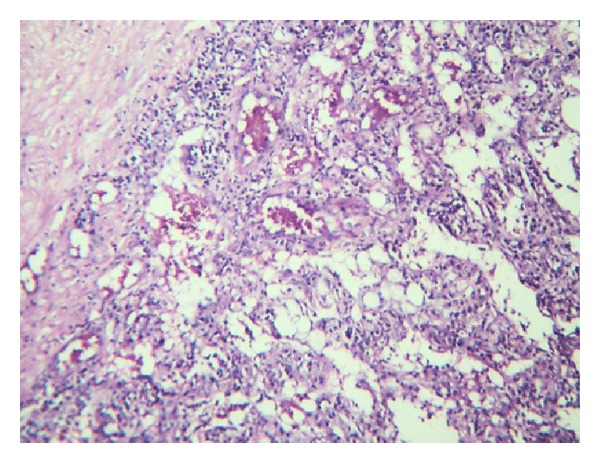
H&E 10x lesion composed of vessels, epithelioid cells, and adipocytes.

**Figure 5:**
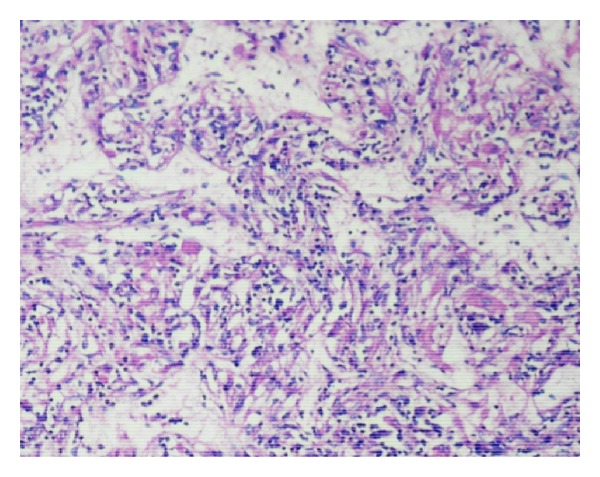
H&E—lesion showing inflammatory infiltrate.

**Figure 6:**
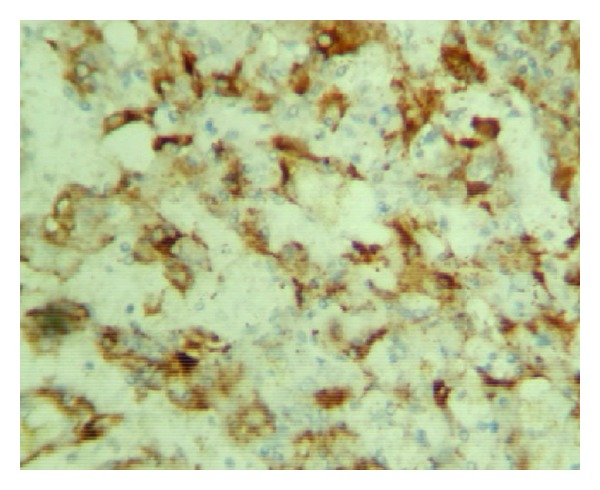
IHC—HMB 45 stain positive in epithelioid cells.

Microscopy showed a neoplasm composed of epithelioid cells, thick-walled blood vessels, and adipocytes. The epithelioid cells had granular pink or vacuolated cytoplasm and vesicular oval/round nuclei, some showing prominent nucleoli. There was pigment in some of the cells. Thick-walled blood vessels were seen dispersed and were prominent near the capsule. Adipocytes were seen in some of the sections focally. Also noted were inflammatory cells comprising of lymphocytes, plasma cells, and histiocytes throughout the lesion (Figures 4 and 5). Extramedullary hematopoiesis and foci of ossification were also noted. The pigments in the cells were found to be melanin and iron by the Masson Fontana and Perl's stain, respectively. Immunohistochemistry was done and the tumor cells were diffusely positive for HMB45 and SMA (Figure 6). The nonneoplastic liver showed mild nonspecific mononuclear infiltration in the portal tracts.

## 3. Discussion

Angiomyolipomas are benign mesenchymal tumors classically described in kidney in association with tuberous sclerosis [1]. Hepatic angiomyolipomas are uncommon. They lead to considerable diagnostic problems clinically, radiologically, and pathologically because of their diverse morphology. Hepatic angiomyolipomas are difficult to diagnose radiologically if the fat content is less [7] as in our case. Macroscopically AMLs are described as solitary, well encapsulated, and soft with a yellow-to-tan or-gray cut surface [7]. Cystic appearance with hemorrhagic and necrotic areas is rare which added to the diagnostic difficulty in our case.

Hepatic angiomyolipomas have been classified histologically depending on the amount of various components that are vessels, smooth muscle cells, and adipocytes as angiolipomas, myolipomas, and angiomyolipomas [8]. Others classify them as lipomatous, myomatous, angiomatous and mixed, amongst which mixed is more common and is composed of equal amounts of above three tissue components [7]. The smooth muscle cells can have an epithelioid, spindle cell, intermediate or pleomorphic morphology [8]. In histology our case showed epithelioid cells which can be confused with a hepatocellular carcinoma [5]. The lipomatous component may be focal as in our case and may add to diagnostic difficulty radiologically. The vascular component consisted of thick- and thin-walled blood vessels which were prominent near the capsule. Five types of blood vessel comprising collagenous, cellular, haemangiopericytic, glomeruloid, and aneurysmal types have recently been described, and it was reported that apart from collagenous vessels, these vessel types were neoplastic components of AML [4]. There were foci of extramedullary hematopoiesis and ossification which are also described [8]. Melanin can be demonstrated in the epithelioid cells by the Masson Fontana stain. Melanin pigment is present in many of the AMLs, and electron microscopic studies have identified premelanosomes in the smooth muscle component [11]. The striking feature in our case was the prominence of inflammatory cells composed of lymphocytes, plasma cells, and histiocytes which were seen throughout interspersed with the other three elements giving the appearance of an inflammatory myofibroblastic tumour. This has been described previously in very few cases and called as inflammatory AMLs when the inflammatory component constituted more than 50% of the tumor [4, 9]. The present case can be included in this category. Immunohistochemistry showed the epithelioid cells to be diffusely positive for HMB45 and SMA which clinched the diagnosis [9].

## 4. Conclusion

This case is reported because of several interesting aspects. Our patient is a 50-year-old male who was thought to have Hepatocellular carcinoma clinically. Radiological differential diagnosis included hepatocellular carcinoma and giant angioma. AMLs are mostly located in the right lobe whereas in our case it was in the left lobe. Grossly the tumour was cystic with necrosis which is unusual in AML. Predominant epithelioid morphology is not common, and the prominent inflammatory component seen in our case has been described only in 11 cases earlier.
